# DISTINCTIVE NEURONAL RESPONSES TO VARIOUS ANTICHOLINERGICS IN THE ACT COHORT HIPSC-DERIVED NEURONS

**DOI:** 10.1093/geroni/igae098.2838

**Published:** 2024-12-31

**Authors:** Inez Pranoto, Tiara Schwarze-Taufiq, Katherine Hui, Ruowei Zhu, Jordan Ogg, Paul Crane, Shelly Gray, Jessica Young

**Affiliations:** University of Washington, Seattle, Washington, United States; University of Washington, Seattle, Washington, United States; University of Washington, Seattle, Washington, United States; University of Washington, Seattle, Washington, United States; University of Washington, Seattle, Washington, United States; University of Washington, Seattle, Washington, United States; University of Washington, Seattle, Washington, United States; University of Washington, Seattle, Washington, United States

## Abstract

Anticholinergic medications remain widely used among older adults despite evidence of safety concerns, including dementia risk. Clinical cohort studies have linked certain anticholinergic classes, such as antidepressants and bladder antimuscarinics, to higher dementia risk. However, distinguishing whether this association stems from individuals’ predisposed conditions or drug-induced neuronal dysfunctions remains challenging. To address this, we used human induced pluripotent stem cell-derived neurons (hiPSC-Ns) from participants in the Adult Changes in Thought Study (ACT) aging study. We treated ACT hiPSC-Ns with four anticholinergic classes: antidepressants, antihistamines, bladder antimuscarinics, and antispasmodics. We evaluated drug-induced neuronal responses at a cellular and molecular level by measuring cytotoxicity, synaptic gene expression, and secreted amyloid-beta, a hallmark of Alzheimer’s disease. We found that exposure to antidepressants and bladder antimuscarinics induced neurotoxicity, while antihistamines and antispasmodics did not compromise cell viability at the concentrations tested. Despite inducing neurotoxicity, antidepressants and bladder antimuscarinic treatments upregulated the expression of various genes involved in synaptic plasticity, synaptic vesicle trafficking/release, and synaptic inhibition. Notably, treatment with a bladder antimuscarinic drug increased the secreted A𝛃-42/A𝛃-40 ratio, indicating a higher production of pathogenic amyloid isoform. Our preliminary findings reveal a consistent elevation in neurotoxicity, synaptic gene expression, and secreted amyloid-beta following exposure to antidepressants and bladder antimuscarinics across hiPSC-Ns from multiple ACT participants. We aim to further investigate the distinct molecular mechanisms of different anticholinergic compounds and their potential impact on declining neuronal functions. Our ongoing investigation provides cellular and molecular evidence to discern the source of dementia risk observed in cohort studies.

